# Germline Mutations Landscape in a Cohort of the State of Minas Gerais, Brazil, in Patients Who Underwent Genetic Counseling for Gynecological and Breast Cancer

**DOI:** 10.1055/s-0042-1757956

**Published:** 2023-03-28

**Authors:** Camila Martins de Carvalho, Letícia da Conceição Braga, Luciana Maria Silva, Anisse Marques Chami, Agnaldo Lopes da Silva Filho

**Affiliations:** 1 Department of Obstetrics and Gynecology, Universidade Federal de Minas Gerais, Belo Horizonte, MG, Brazil; 2 OncoTag Desenvolvimento de Produtos e Serviços para Saúde Humana, Belo Horizonte, MG, Brazil; 3 Translational Research Laboratory in Oncology, Instituto Mário Penna-Ensino, Pesquisa e Inovação, Belo Horizonte, MG, Brazil; 4 Cell Biology Service, Diretoria de Pesquisa e Desenvolvimento, Fundação Ezequiel Dias, Belo Horizonte, MG, Brazil; 5 School of Medicine, Campus Botucatu, Universidade Estadual Paulista, Belo Horizonte, MG, Brazil

**Keywords:** germline variants, genetic counseling, gynecological cancer risk, hereditary syndromes, variantes de linhagem germinativa, aconselhamento genético, risco de câncer ginecológico, síndromes hereditárias

## Abstract

**Objective** The present study evaluated the profile of germline mutations present in patients who underwent genetic counseling for risk assessment for breast cancer (BC), ovarian cancer (OC), and endometrial cancer (EC) with a possible hereditary pattern.

**Methods** Medical records of 382 patients who underwent genetic counseling after signing an informed consent form were analyzed. A total of 55.76% of patients (213/382) were symptomatic (personal history of cancer), and 44.24% (169/382) were asymptomatic (absence of the disease). The variables analyzed were age, sex, place of birth, personal or family history of BC, OC, EC, as well as other types of cancer associated with hereditary syndromes. The Human Genome Variation Society (HGVS) nomenclature guidelines were used to name the variants, and their biological significance was determined by comparing 11 databases.

**Results** We identified 53 distinct mutations: 29 pathogenic variants, 13 variants of undetermined significance (VUS), and 11 benign. The most frequent mutations were *BRCA1* c.470_471delCT, *BRCA1* c.4675 + 1G > T, and *BRCA2* c.2T> G. Furthermore, 21 variants appear to have been described for the first time in Brazil. In addition to *BRCA1/2* mutations, variants in other genes related to hereditary syndromes that predispose to gynecological cancers were found.

**Conclusion** This study allowed a deeper understanding of the main mutations identified in families in the state of Minas Gerais and demonstrates the need to assess the family history of non-gynecological cancer for risk assessment of BC, OC, and EC. Moreover, it is an effort that contributes to population studies to evaluate the cancer risk mutation profile in Brazil.

## Resumo

**Objetivo** O presente estudo avaliou o perfil de mutações germinativas presentes em pacientes submetidas a aconselhamento genético para avaliação de risco para câncer de mama (CM), câncer de ovário (OC) e câncer de endométrio (CE) com possível padrão hereditário.

**Métodos** Foram analisados os prontuários de 382 pacientes que realizaram aconselhamento genético após consentimento informado. Um total de 55,76% dos pacientes (213/382) eram sintomáticos (história pessoal de câncer), e 44,24% (169/382) eram assintomáticos (ausência da doença). As variáveis analisadas foram idade, sexo, naturalidade, história pessoal ou familiar de CM, OC, CE bem como outros tipos de câncer associados a síndromes hereditárias. As diretrizes de nomenclatura da Human Genome Variation Society (HGVS) foram usadas para nomear as variantes e seu significado biológico foi determinado pela comparação de 11 bancos de dados.

**Resultados** Identificamos 53 mutações distintas: 29 variantes patogênicas, 13 variantes de significado indeterminado e 11 benignas. As mutações mais frequentes foram BRCA1 c.470_471delCT, BRCA1 c.4675 + 1G > T e BRCA2 c.2T > G. Além disso, 21 variantes parecem ter sido descritas pela primeira vez no Brasil. Além das mutações BRCA1/2, foram encontradas variantes em outros genes relacionados a síndromes hereditárias que predispõem a cânceres ginecológicos.

**Conclusão** Este estudo permitiu conhecer melhor as principais mutações identificadas nas famílias do estado de Minas Gerais e demonstra a necessidade de avaliar a história familiar de câncer não ginecológico para avaliação do risco de CM, OC e CE. Além disso, é um esforço que contribui com estudos populacionais para avaliar o perfil de mutações de risco para câncer no Brasil.

## Introduction

Breast cancer (BC) is the most common type of cancer in women, excluding non-melanoma skin cancer. In 2020, there were almost 2.3 million new cases of BC worldwide, representing 24.5% of all cancer cases in women. 1 On the other hand, ovarian cancer (OC) is the most lethal gynecological malignancy. In 2020, there were 313,959.414 OC cases and 207,252 deaths from it. 1 Endometrial cancer (EC) is the 6 ^th^ most common type of cancer in women, with a worldwide incidence in 2020 of 417,367 and 97,370 deaths. 1 In Brazil, for the 2020 to 2022 biennium, the rate of new cases of OC, BC, and EC is 6,650; 66,280 and 6,540 per year, respectively. 2 In Minas Gerais, 8,250 cases of BC, 630 of OC, and 670 of EC were estimated in 2020. 2

Most gynecological cancers are sporadic, but ∼ 5% of EC, 25% of OC, and 10 to 30% of BC have a hereditary pattern. 3 4 Most cases of hereditary BC and OC are attributable to mutations in one of the *BRCA* genes, which also increase the risk of other cancers. 3

Hereditary tumor-associated syndromes such as Lynch syndrome (LS), Li-Fraumeni syndrome (LFS), Peutz-Jeghers syndrome (PJS), and Cowden syndrome (CS) also represent an important feature in the carcinogenesis of gynecological and breast tumors. Lynch syndrome is an autosomal dominant inherited syndrome associated with a mutation in one or more mismatch repair (MMR) pathway genes ( *MSH2, MLH1, MSH6,* and *PMS2* ) or in the *EPCAM* gene, which is *MSH2* 's regulator. About 15% of OC cases and 2 to 6% of EC cases are caused by LS. 5 Li-Fraumeni syndrome is associated with *TP53* germline mutations, determining a high risk of developing primary cancers. 6 Peutz-Jeghers syndrome is a rare disorder associated with pathogenic mutations in *STK11* . This syndrome confers an elevated lifetime risk of several cancers as gastrointestinal, BC and OC. 7 Cowden syndrome is a rare but clinically diagnosable multiple hamartoma syndrome, and it is associated with *PTEN* germline mutations. Cowden syndrome confers up to 85% lifetime risk of BC and up 30% of EC. 8

Other genetic variants also predispose to the neoplasm risk, such as *PALB2* (high risk associated), and *CHEK2, ATM, BARD1,* and *RAD51D* (moderate risk associated). 9 *RAD51C, RAD51D,* and *BRIP1* mutations were also related to increased OC risk. 9

The understanding of cancer genetic predisposition leads to better identification of patients at risk; thus, physicians will be able to coordinate strategies for detection, management, and prevention. 10 This study aims to evaluate the germline mutations profile in patients from different regions of Minas Gerais state, who were submitted to genetic counseling for risk assessment for BC, OC, and EC with possible hereditary patterns.

## Methods

Between April 2017 and October 2018, a cohort of 382 patients undergoing genetic counseling due to suspected hereditary cancer at a private genetic referral center in Belo Horizonte was analyzed. In general, the main syndromes have gynecological cancer as part of the also main phenotype, as explained in the introduction. This study was approved by the Research Ethics Committee of Universidade Federal de Minas Gerais (CAEE: 01758418.0.0000.5149), and prior written consent was accepted from all participants. Age, gender, naturality, personal or family history of BC, OC, EC, and other cancers associated with hereditary syndromes as well as genetics tests and their outcome were analyzed. Naturality was defined according to the mesoregions of Minas Gerais, in agreement with the Brazilian Institute of Geography and Statistics (IBGE). The criteria for genetic testing followed the National Comprehensive Cancer Network (NCCN) 11 guideline for genetic/familial high-risk assessment, according to the time when the study was performed, since NCCN guideline is actualized constantly. Thus, considering the hereditary cancer predisposition syndromes, many other cancers were considered as part of the family history phenotype. Although all patients received pretest genetic counseling, the test methodology chosen varied according to the test availability for each patient. Since it was a private service, most of the patients underwent genetic tests following the approval of their current health plan or they paid for it on their own. This means that some patients only had access to tests related to *BRCA1* and *BRCA2* genes, for example, and others have performed commercial panels for hereditary cancer. The Human Genome Variation Society (HGVS) nomenclature guidelines ( http://varnomen.hgvs.org/ ) were used to name the variants. The biological significance of variants reported was assessed in the ClinVar ( www.ncbi.nlm.nih.gov/clinvar/ ), Brazilian Genomic Variants (ABraOM - http://abraom.ib.usp.br/ ), dbSNP ( https://www.ncbi.nlm.nih.gov/snp/ ), European Variant Archive (EVA - https://www.ebi.ac.uk/eva ), GeneCards ( https://www.genecards.org/ ), GnomAD. ( https://gnomad.broadinstitute.org/variant ), The Human Gene Mutation Database ( http://www.hgmd.cf.ac.uk ), UniProt ( https://www.uniprot.org/ ), Varsome ( https://varsome.com/variant ), 1000 genomes ( https://www.ncbi.nlm.nih.gov/variation/tools/1000genomes/ ), and BRCA Exchange ( https://brcaexchange.org/ ) databases.

## Results

**Fig. 1:**
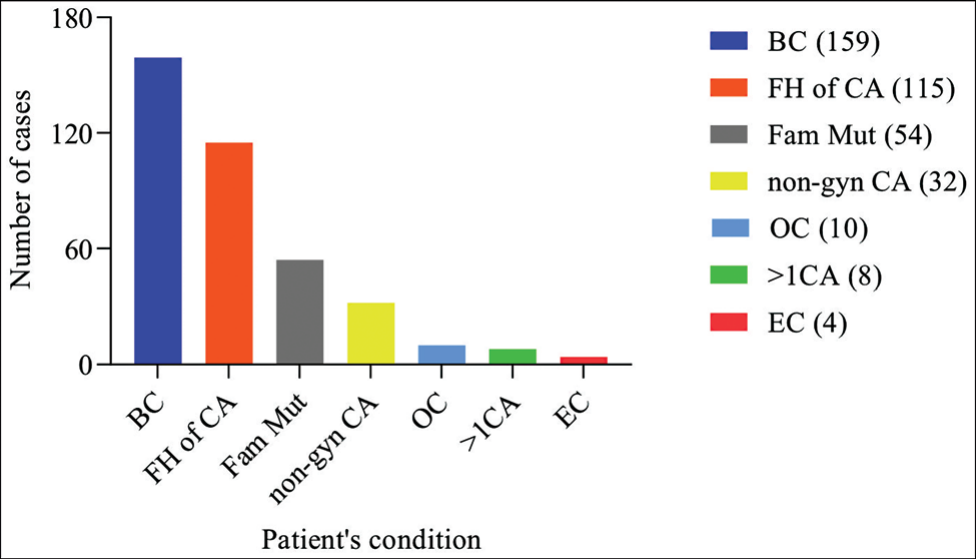
Reason for medical consultation. BC, personal history of breast cancer; FH CA, family history of cancer without previously identified mutation; FamMut, identified family mutation; non-gyn CA, personal history of non-gynecological cancer associated with hereditary syndromes; OC, personal history of ovarian cancer; >1CA, personal history of more than one type of cancer; EC, personal history of endometrial cancer.

Three hundred eighty-two patients with personal and/or family history of BC, OC, EC, and other cancers associated with hereditary syndromes were selected at a referral center in Belo Horizonte, Minas Gerais. Three hundred fifty-four patients were female (354/382, 92.7%) and 28 were male (28/382, 7.3%). A total of 55.76% of patients (213/382) were considered symptomatic, and 44.24% (169/382) were considered asymptomatic ( Fig. 1 ).

**Fig. 2:**
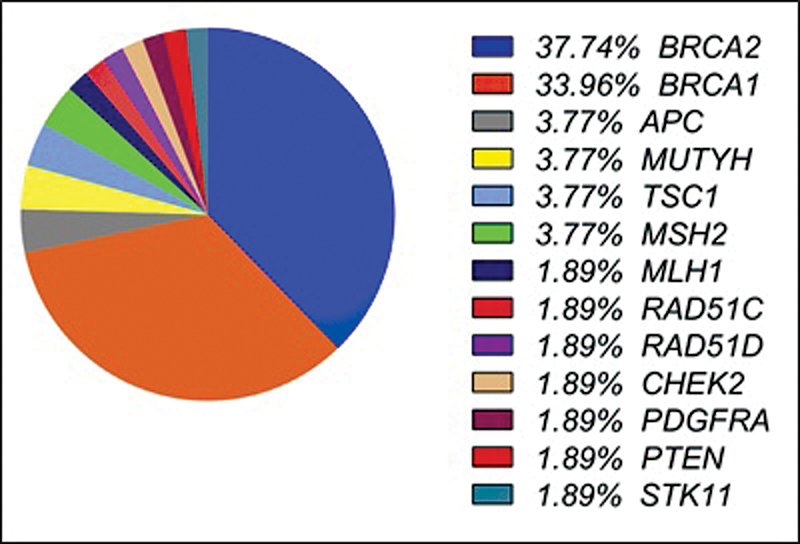
Percentage of distinct variants detected in each gene evaluated.

Of the symptomatic patients, 159 had a personal history of BC, 10 of OC, 4 of EC, 32 were diagnosed with non-gynecological cancers associated with hereditary syndromes, and 8 patients had more than one type of cancer: 1 had BC and OC associated, 1 BC and EC, 1 BC, OC, and melanoma, and 5 had BC and non-gynecological cancers. Among the asymptomatic patients, 54 sought genetic counseling due to previously identified family mutation and 115 due to a family history of cancer without previously identified mutation, and only 49.7% (84/169) were eligible for genetic tests. There were no patients younger than 18 years (0/382), according to the exclusion criteria, 1 between 18 and 20 years (1/382, 0.26%), 45 between 21 and 30 years (45/382, 11.78%), 113 between 31 and 40 years (113/382, 29.58%), 101 between 41 and 50 years (101/382, 26.44%), 64 between 51 and 60 years (64/382, 16.75%), 43 aged 61 to 70 years (43/382, 11.26%), 8 aged over 70 years (8/382, 2.10%), and there were no data on the age of 7 patients (7/382, 1.83%). Most patients were from Belo Horizonte Metropolitan Region (277/382), followed by West of Minas Gerais, with 21/382 patients, 18/382 from the Rio Doce Valley, 10/382 from Campo das Vertentes, 9/382 from the North of Minas Gerais, 8/382 from the Central Region, 8/382 from Zona da Mata, 4/382 from South/Southwest of Minas Gerais, 3/382 from Mucuri Valley, 2/382 from Triângulo Mineiro/Alto do Parnaíba, and 2/382 from Jequitinhonha. There were no cases from the Northwest of Minas Gerais. Three cases had Ashkenazi ancestry, all from the Belo Horizonte Metropolitan Region, but 1 with family from Poland, 1 from Turkey, and 1 from Romania. Four cases were from other countries: 3 from Lebanon and 1 from Italy. There were no data in the medical records about the place of birth of 5 patients. A total of 85 variants were identified in 72 patients. Among these, 50% had a personal history of BC (36/72), 1 of which was associated with EC, and 1 associated with a non-gynecological cancer, 29 were asymptomatic (29/72), 5 had previous history of non-gynecological cancers (5/72), and 2 had a personal history of OC (2/72). Of the 29 asymptomatic patients, 23 were patients with a family history of previously identified mutation and 6 had a family history of cancer, with no previously identified mutation. Of the 15/72 patients with variants in other genes, excluding *BRCA1* /2, 5 were diagnosed with BC, 1 with BC and EC, 10 were asymptomatic or diagnosed with non-gynecological cancer, but all had a family history of BC, and 1 asymptomatic patient had a family history of OC. The age at diagnosis of BC in these patients ranged from 33 to 64 years. Of these, there were 53 distinct mutations: 20 in *BRCA2* , 18 in *BRCA1* , 2 in *APC* , 2 in *MUTYH* , 2 in *TSC1* , 2 in *MSH2* , 1 in *MLH1* , 1 in *RAD51D* , 1 in *CHEK2* , 1 in *PDGFRA* , 1 in *PTEN* , and 1 in *STK11* ( Fig. 2 ) ( Table 1 ).

**Table 1:** Different variants identified in the cohort study

P/LP	VUS	B/LB
APC del éxons 17–18 (1)	BRCA1 c.1713A > G (1)	APC c.5465T > A (1)
BRCA1 c.2037delinsCC (1)	BRCA1 c.220C > A (1)	BRCA1 c.804G > A (1)
BRCA1 c.211A > G (1)	BRCA2 c.1146A > T (1)	**BRCA1 c.-19–115 T** **>** **C (2)**
**BRCA1 c.3331_3334delCAAG (2)**	BRCA2 c.3196A > C (1)	BRCA1 c.2612C > T (1)
BRCA1 c.4675 + 1G > A (1)	BRCA2 c.5096A > G (1)	BRCA1 c.1486C > T (1)
**BRCA1 c.4675** **+** **1G** **>** **T (6)**	BRCA2 c.6988A > G (1)	**BRCA2 c.7397 C** **>** **T (2)**
**BRCA1 c.470_471delCT (7)**	BRCA2 c.8305G > C (1)	**BRCA2 c.7806–14 T** **>** **C (2)**
BRCA1 c.4964_4982del (1)	BRCA2 c.640G > A (1)	**BRCA2 c.8755–66 T** **>** **C (2)**
**BRCA1 c.5266dupC (3)**	CHEK2 c.319 + 3966G > A (1)	MUTYH c.1014G > C (1)
**BRCA1 c.5467** **+** **3A** **>** **C (2)**	PDGFRA c.718A > C (1)	STK11 c.1038C > T * (1)
**BRCA1 c.798_799delTT (3)**	RAD51C c.428A > G (1)	TSC1 c.625A > G (1)
BRCA1 del éxons 18–19 (1)	RAD51D c.26G > C (1)	
BRCA1 c.4964C > T (1)	TSC1 c.3301G > A (1)	
**BRCA2 c.6591_6592del (4)**		
BRCA2 c.156_157insAlu (1)		
**BRCA2 c.1796_1800delCTTAT (2)**		
BRCA2 c.2978G > A (1)		
**BRCA2 c.2T** **>** **G (5)**		
BRCA2 c.4829_4830delTG (1)		
BRCA2 c.5985delC (1)		
BRCA2 c.6275_6276del (1)		
**BRCA2 c.6405_6409delCTTAA (2)**		
BRCA2 c.7819_7819delA (1)		
**BRCA2 c.9154C** **>** **T (3)**		
MLH1 del éxons 17, 18 e 19 (1)		
MSH2 c.1894_1898del (1)		
MSH2 c.2152C > T (1)		
MUTYH c.536A > G (1)		
PTEN c.264T > G (1)		

Abbreviations: (#), number of probands; B/LB, benign/likely benign; P/LP, pathogenic/likely pathogenic; VUS, variant of uncertain significance.

* This variant has 4 classifications VUS and 4 Benign. Variants in bold were identified in more than one patient.

**Fig. 3:**
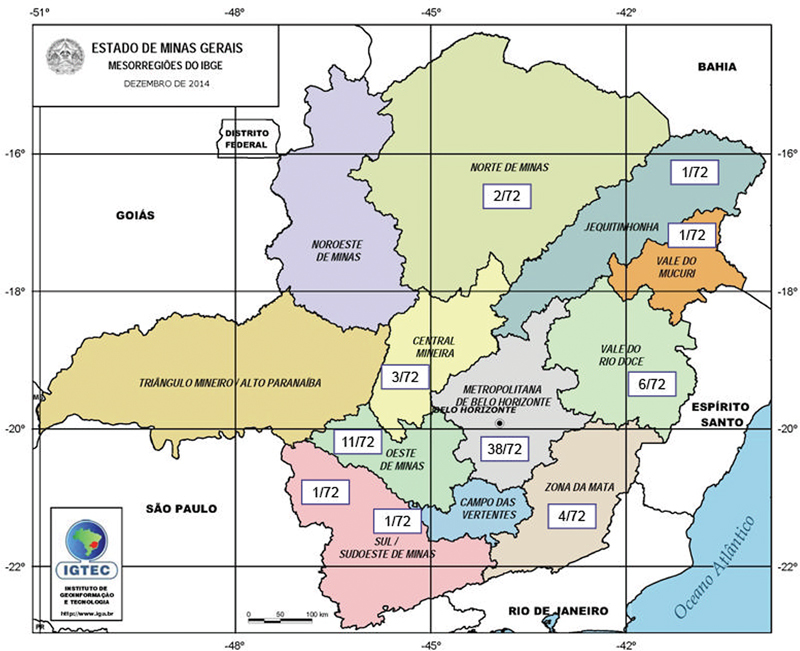
Geographical distribution of variants identified in Minas Gerais State mesoregions, according to the Brazilian Institute of Geography and Statistics (IBGE). Two patients were from other countries, and one from another state. There were no patients from the Northwest and Triângulo Mineiro regions.

The most frequent mutation was *BRCA1* c.470_471delCT, which appeared in 7 cases (7/53) in 5 distinct families, followed by *BRCA1* c.4675 + 1G > T, with 6 cases (6/53), 5 of which were in the same family, and *BRCA2* c.2T > G, with 5 cases (5/53) identified in 4 distinct families. Five variants ( *BRCA1* c.1713A > G, *BRCA2* c.3196A > C, c.5096A > G, c.6988A > G, and *RAD51C* c.428A > G) are considered as VUS in ClinVar, and other tools were re-classified as pathogenic in Varsome and the *RAD51D* c.26G > C was changed to benign variant. The following variants appear to have been described for the first time in Brazil: *APC* c.5465T > A, *BRCA1* c.1713A > G, c.220C > A, c.804G > A, c.5467 + 3A > C,; *BRCA2* c.5985delC, c.7819_7819delA, c.6591_6592delTG, c.1146A > T, c.2978G > A, c.3196A > C, c.6275_6276del, c.640G > A, c.8305G > C, *CHEK2* c.319 + 3966G > A, *MSH2* c.1894_1898del, *PDGFRA* c.718A > C, *PTEN* c.246T > G, *RAD51C* c.428A > G, *TSC1* c.3301G > A, and *TSC1* c.625A > G. Of the 72 patients who had mutations identified, the majority was from the Belo Horizonte Metropolitan Region (38/72) followed by West of Minas Gerais, with 11/72 patients, 6/72 from the Rio Doce Valley, 4/72 from Zona da Mata, 3/72 from the Central Region, 2/72 from the North of Minas Gerais, and, finally, South/Southwest of Minas Gerais, Campo das Vertentes, Mucuri Valley, and Jequitinhonha with 1 patient each ( Fig. 3 ).

There were no patients from the Northwest and Triângulo Mineiro regions. Two patients were from other countries and one from another state. None of these patients declared Ashkenazi ancestry.

## Discussion

Genetic testing for patients with a high risk for gynecological cancer enables cancer risk reduction strategies, such as salpingo-oophorectomy and mastectomy, 12 chemoprevention, and specific therapeutics, such as PARP inhibitors in BRCA-mutated patients. 13 Besides, it allows differentiated cancer screening for early detection, such as breast magnetic resonance image and mammography at an early age. 13 Among patients in the United States, for whom *BRCA1* and *BRCA2* mutation tests have become universal in clinical practice for OC patients, a reduction of 40% is estimated in the incidence of OC and 37 to 64% of BC in 10 years in healthy family members diagnosed with pathogenic mutation. 14

It is necessary to identify the most common mutations in each population to develop a specific panel, thus making the process more efficient and less costly. So, mutation frequency studies should be conducted. 15 For the self-declared Ashkenazi patients, 3 founder mutations in *BRCA* ( *BRCA1* c.5266dupC, *BRCA1* c.68_69del, and *BRCA2* c.5946del) correspond to 98 to 99% of mutations identified. 16 17

The Brazilian population is one of the most heterogeneous in the world. 17 Then, due to a lack of local studies, all recommendations are based on international data. One of the major strengths of this study is that it is the first to evaluate the germline mutations profile in the state of Minas Gerais.

The most frequent mutation found in this study was *BRCA1* c.470_471delCT, which differs from those already performed in Brazil, in which *BRCA1* c.5266dupC was the most common. 15 17 The *BRCA1* c.470_471delCT mutation has been reported in 49 studies in the BRCA Exchange database, and it is the most prevalent mutation in Hong Kong, 15 Malaysia, Southeast China, 18 Japan, 19 and Spain. 20 *BRCA1* c.470_471delCT was identified in 5 different families, 2 of which are native from Vale do Rio Doce. One possible explanation is the beginning of commercial exploratory activities between Japan and Brazil, in 1950, in this region. Another is a founder mutation there. Of the 7 cases identified with this mutation, 5 had BC, with 3 of them under the age of 40, and 1 had OC at 36 years old.

The most prevalent mutation in *BRCA2* was c.2T > G, as previously described, 17 21 and it was present in 5 patients from 4 different families. Among these patients, 3 were diagnosed with BC, 2 of them younger than 40 years old. One patient diagnosed at 33 years old had disease recurrence. In all cases, family history is significant for BC and PC, which demonstrates the importance of the genetic test for predictive medicine in gynecological oncology.

*BRCA1* c.5266dupC is a founder mutation in Ashkenazi, and it is one of the most frequent mutations worldwide, including in Brazil. 15 However, three cases were identified, none reported as Ashkenazi ancestry.

The Portuguese founder mutation *BRCA2* c.156_157insAlu corresponds to more than a quarter of the *BRCA1/2* mutations found in Portugal 20 and in Brazil, and it was frequently in Palmero's study. 17 Although Brazil received more than 2 million Portuguese between 1500 and 1991, this variant had a low prevalence in other studies. 22 In our study, the mutation was found in only one patient. This mutation may not have been identified in the tests performed due to low prevalence or because it is a large insertion and may require a specific PCR-based test. However, all patients are advised to keep their follow-up at the medical genetics service up to date and review their clinical and laboratory data.

Mutations prevalent in other studies in Brazil, such as *BRCA1* c.3331_3334delCAAG 17 and *BRCA1* c.211A > G, 17 were found in 2 and 1 patients, respectively. In *BRCA2* , the frequent variants *BRCA2* c.2808_2811delACAA and *BRCA2* c.5946ddelT, previously described, 17 21 were not identified in our cohort.

Another strength of our study is the evaluation of other genes involved in the predisposition to gynecological cancers. In southern and southeastern Brazil, the founder mutation *TP53* c.1010G > A (p.R337H) was identified in 0.3% of the population, 23 which corresponds to 300 times more than any LFS-associated mutation. 24 Although a search for this specific mutation was requested for 11 (15.3%) patients with suspected LFS or LFL in our cohort, none was identified as well as in one study performed in Belo Horizonte. 25 This information makes us question what the real prevalence of this mutation in Minas Gerais population is. Are the R337H carriers referred for genetic counseling? And what is their real cancer risk?

We identified one patient diagnosed with a pathogenic mutation in *APC* without personal cancer history but with BC and colorectal cancer (CRC) family history. Three patients were diagnosed with LS: one had EC at 54 years old and BC at 55 years old, besides a family history of BC, CRC, pancreas cancer, and melanoma; one was asymptomatic with OC, PC, CRC, and leukemia family history, and the other one had CRC at 51 years old and has a family history of CRC and pancreas cancer.

The diagnosis of LS in patients and their families is extremely important, due to the high risk of developing EC and OC and the evaluation of possible risk reduction management. The 3 mutations identified here are pathogenic, two in *MSH2 (MSH2* c.1894_1898del and *MSH2* c.2152C > T) and 1 in *MLH1 (MLH1* del exons 17, 18, and 19). The patient diagnosed with the pathogenic mutation *MUTYH* c.536A > G had no cancer personal history but had a family history of BC, CRC, PC, and sarcoma. *MUTYH* mutations are associated with an elevated risk of CRC, EC, and BC. 26 27

One patient with CS diagnostic and pathogenic mutation *PTEN* c.264T > G was also diagnosed with thyroid cancer at 24 years old. Although, she has no family history of gynecological cancers, it is important to identify this mutation due to the high risk of developing BC and EC.

In this study, 13 VUS were found: 2 in *BRCA1* , 6 in *BRCA2,* and 1 in *CHEK2* , *PDGFRA* , *RAD51C* , *RAD51D,* and *TSC1* , each. Variants of undetermined significance is a gene sequence alteration with an unknown consequence on the gene function. 28 Counseling patients with VUS results is challenging for at least two reasons. First, it does not estimate the cancer risk and, therefore, does not allow guidance on preventative measures. Secondly, the variant reclassification is a dynamic process and needs great attention to patients' care. Initially, VUS must be treated as negative, and the risk assessment should be based on family history. 29

In this study, the patients were selected from a private genetic center, which limits our study as most of the Brazilian population depends on the public health system. Furthermore, genetic testing coverage by health plans determines the difference in the methodology used between patients. Nevertheless, it allowed the evaluation of a patient population undergoing genetic counseling for hereditary cancer within the reality of clinical practice.

## Conclusion

Considering the impact of a pathogenic/likely pathogenic mutation on the patient and their family members, it is important to understand these population genetic profiles to offer better genotype-phenotype correlation to guide clinical decisions and effective management to reduce the cancer risk in a more democratic way which is adaptable to health care.
